# Quantifying Effect of Geographic Location on Epidemiology of *Plasmodium vivax* Malaria

**DOI:** 10.3201/eid1907.121674

**Published:** 2013-07

**Authors:** Andrew A. Lover, Richard J. Coker

**Affiliations:** Saw Swee Hock School of Public Health, National University of Singapore, Singapore (A.A. Lover, R.J. Coker);; London School of Tropical Medicine and Hygiene, Bangkok, Thailand (R.J. Coker)

**Keywords:** Plasmodium vivax, vivax, geographic location, survival analysis, population surveillance, epidemiology, temperate, tropical, malaria, parasites, vector-borne infections

## Abstract

Recent autochthonous transmission of *Plasmodium vivax* malaria in previously malaria-free temperate regions has generated renewed interest in the epidemiology of this disease. Accurate estimates of the incubation period and time to relapse are required for effective malaria surveillance; however, this information is currently lacking. By using historical data from experimental human infections with diverse *P. vivax* strains, survival analysis models were used to obtain quantitative estimates of the incubation period and time to first relapse for *P. vivax* malaria in broad geographic regions. Results show that Eurasian strains from temperate regions have longer incubation periods, and Western Hemisphere strains from tropical and temperate regions have longer times to relapse compared with Eastern Hemisphere strains. The diversity in these estimates of key epidemiologic parameters for *P. vivax* supports the need for elucidating local epidemiology to inform clinical follow-up and to build an evidence base toward global elimination of malaria.

The malaria parasite *Plasmodium vivax*, which received limited research attention for a number of decades, has moved onto the global health agenda for 2 key reasons. First, compared with the *P. falciparum* parasite, which also causes malaria, the *P. vivax* parasite is more difficult to eliminate because it has a broader geographic range and, unlike *P. falciparum*, has dormant liver stages. Second, *P. vivax* malaria has reemerged in previously malaria-free temperate regions, including Greece, Corsica, the Korean Peninsula, central China, and Australia (*1*–*5*). Moreover, increasing evidence indicates that *P. vivax* infections can be severe and fatal (*6*,*7*).

A large body of epidemiologic and clinical data supports the existence of discrete strains within each *Plasmodium* species. Much of the reliable data are from patients who were infected in temperate climates (study centers in the United States, United Kingdom, and Europe), and the parasites were from various locales. Tropical vivax strains generally cause a larger number of closely spaced relapses, and temperate strains have generally evolved to cause a long incubation period, enabling the survival of the parasite as dormant hypnozoites during colder months (*8*,*9*). Malariologists have long recognized that the incubation period for malaria varies by strain and geographic latitude (*10*).

Recent studies of temperate *P. vivax* strains in South Korea support these observations and suggest that chemoprophylaxis of infected patients may contribute to long latency (*11*,*12*). However, these observational studies could not estimate the time from infection to relapse or relapse periodicity because exact infection times were unknown. In a related primaquine dosing study, the relapse rate for 3 vivax strains was compared, but relapse time was not examined (*13*).

Numerous *P. vivax* classification schemes have been suggested, including temperate/tropical, temperate/subtropical/temperate, and northern/southern/Chesson-type. These schemes were suggested on the basis of observed clinical characteristics, but quantitative data to support these distinctions are sparse (*14*–*16*). Recent molecular and entomologic data suggests that *P. vivax* may consist of 2 subspecies, 1 in the Old World/Eastern Hemisphere (*P. vivax vivax)* and the other in the Americas (*P. vivax collins*) (*17*); however, research with other isolates has not confirmed these results (*18*). These 2 strains/subspecies show remarkable differences in their infectivity to different *Anopheles* spp. mosquitoes; however, the effect of these differences on the epidemiology of malaria in humans has not been demonstrated. In addition, at least 2 other subspecies, *P. vivax hibernans* and *P. vivax multinucleatum*, produce disease that is similar to currently circulating strains from the Korean Peninsula (*19*,*20*). 

A large body of historical data exists from the deliberate laboratory infection of 2 populations: 1) patients receiving malariotherapy (intentionally induced malaria) for neurosyphilis and related disorders; and 2) healthy prisoners who participated in malaria drug testing trials. We used the experimental infection data from these controlled settings to explore and quantify the relationship between *P. vivax* parasite origin and characteristics of malaria caused by these subpopulations.

## Materials and Methods

### Data Sources and Selection Criteria

#### Search Strategy

We performed a comprehensive literature search of MedLine and Google Scholar in English, searching for (“vivax” OR “benign tertian”) AND (“induced” OR “human” OR “experimental”). Limited searches were performed in Dutch, German, and French. The citations in these initial papers were examined, resulting in the identification of a large number of non-indexed papers.

#### Data Inclusion Criteria

We included data for experimentally infected persons who 1) were malaria naive before the experimental infection, 2) had defined inoculation dates (infection by mosquito only; persons with infections from sporozoite injection or blood transfer were excluded), 3) had received only well-documented (or explicit) treatment of symptoms, 4) were protected against reinfection, and 5) were infected with traceable strains that had defined origins. Studies that measured prepatent period (i.e., the time to first appearance of blood-stage parasites, seen by microscopy) were excluded. Data for patients with incubation periods >50 days were excluded from the analysis because only 7 such cases were found in well-defined studies. For the infection relapse study, we included data only from studies that had unambiguous follow-up periods. Available covariates were extracted from these studies, and the individual records were digitized by using Plot Digitizer (http://plotdigitizer.sourceforge.net), as needed, to create individual case-patient records. 

Our incubation period analyses included data for 453 patients (infected with a total of 11 strains) from 19 studies (Table 1); data for 6 of the patients were excluded because of highly outlying covariate patterns (Technical Appendix). The first relapse analyses included data for 320 patients (infected with a total of 18 strains) from 15 studies (Table 1); data for 4 of the patients were excluded because of highly outlying covariate patterns (Technical Appendix). Details of the parasite strains are shown in Table 2. Age and sex were not recorded for most patients who had neurosyphilis. All prison volunteers were White men. Several sources had interval censoring; that is, infections were reported as occurring within 1 month or 16 weeks of the mosquito bite. To facilitate comparisons between studies that reported relapse times by integer weeks, relapse dates reported as exact days were analyzed as the next full week from infection. The final relapse event in these data occurred 65 weeks after initial infection; longer follow-up times were right-censored at 78 weeks from initial infection. Latitude of parasite isolation was determined from the site of isolation in the original reports and coded as a binary variable (dividing at ±23.5° South/North latitude) for determining tropical and temperate *P. vivax* parasites. In our analyses, New World strains are those from the Americas, and Old World strains are those from Eurasia, Africa, and the Pacific region, as suggested by Li et al. (*17*).

**Table 1:** Infection data recorded for 677 case-patients in historical studies of *Plasmodium vivax* infection, circa 1920–1980*

Infection data	No. (%) patients
Neurosyphilis/neurologic treatment patient	433 (64.0)
Recorded data	
Exact incubation period only	356 (52.6)
Time to relapse only	217 (32.1)
Exact incubation and time to relapse	104 (15.4)
Infected with	
Tropical strains	275 (40.6)
Temperate strains	402 (59.4)
New World strains	181 (26.7)
Old World strains	496 (73.3)

*The study population was followed for a mean of 82.5 weeks (SD 31.7, range 2.0–173.0).

**Table 2:** *Plasmodium vivax* strains included in a study quantifying the effect of geographic location on the epidemiology of *Plasmodium vivax* malaria

Strain	Place of origin, date	No. case-patients	Malariotherapy	% case-patients given malariotherapy	Region of origin
Chesson	Papua New Guinea, circa 1944	145	No		Tropical
Hlebnikovo	Moscow Oblast, 1948	19	Yes	100.0	Temperate
Holland	Netherlands, circa 1928	52	Yes	100.0	Temperate
Korea	North Korea, 1953	21	Yes	100.0	Temperate
Leninabad	Tajikistan, 1950	33	Yes	100.0	Temperate
Madagascar	Madagascar, 1925	83	Yes	100.0	Tropical
McCoy	Florida, USA, 1931	70	Yes	100.0	Temperate
Moscow	Moscow, 1950	55	Yes	100.0	Temperate
NICA (Nicaragua)	Nicaragua, circa 1970	6	No		Tropical
Nahicevan	Azerbaijan, 1937	5	Yes	100.0	Temperate
Naro-Fominsk	Moscow Oblast, 1946	21	Yes	100.0	Temperate
Panama	Panama, circa 1970	10	No		Tropical
Rjazan	Ryazan, Russia circa 1945	21	Yes	100.0	Temperate
St. Elizabeth	Southern USA, circa 1925	73	Yes	34.2	Temperate
Salvador I	El Salvador, circa 1970	11	No		Tropical
Salvador II	El Salvador, circa 1970	11	No		Tropical
South Vietnam	Southern Vietnam, circa 1972	5	No		Tropical
Vietnam (North)	Northern Vietnam, 1954	4	Yes	100.0	Tropical
Volgograd	Volgograd, Russia, 1945	24	Yes	100.0	Temperate
West Pakistan	Pakistan, 1968	5	No		Temperate
*P. vivax multinucleatum*	Central China, 1965	3	No		Temperate
Total		677		64.0	

### Statistical Analyses

Kaplan-Meir analysis was used to examine the unadjusted relationship between parasite origin and event times; differences were assessed by using a log-rank test. Multivariate models were used to overcome limitations of Kaplan-Meier analyses by allowing for adjustment for the effect of malariotherapy and by producing hazard ratios (HRs) to gauge the strength of association. The standard Cox proportional hazards model cannot provide confidence intervals for predicted survival times; therefore, the more complex, flexible parametric Royston-Parmar models were used to provide covariate-adjusted HRs and covariate-adjusted median survival times for parasite subpopulations (*21*). These models extend Cox methods by adding parameters that model the underlying hazard of a disease event, which allows for more comprehensive predictions. To provide estimates that are relevant to natural malaria infections in humans, the predicted survival times from this analysis were made for a population not receiving treatment for neurologic symptoms. 

The incubation period and time-to-relapse models include the geographic region and neurologic treatment status as covariates for survival time; for the time-to-relapse models, geographic region was modeled as a time-varying covariate due to proportional hazard violations (the effect of regions is allowed to vary through follow-up time). Both models also incorporate adjustments for intragroup correlation caused by the clustering of effects among persons infected with the same parasite strain. 

We used Stata 12.1 (StataCorp, College Station, TX, USA) to perform statistical analyses; all tests were 2-tailed. Detailed methods are in the online Technical Appendix. 

## Results

### Incubation Period

**Figure 1:**
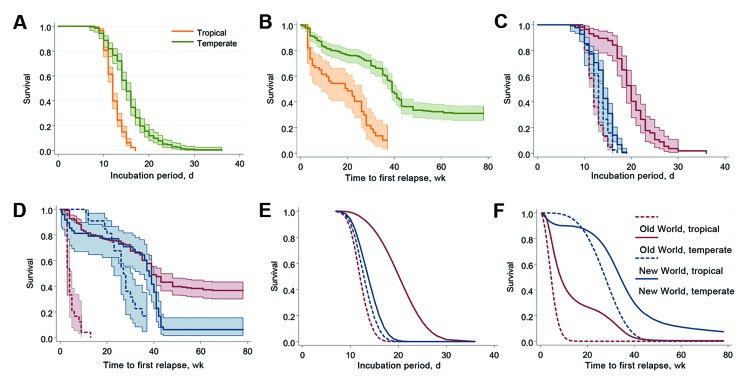
Various modeling estimates of incubation period and time to first relapse for *Plasmodium vivax* malaria in a study quantifying the effect of geographic location on the epidemiology of the infection. A) Kaplan-Meier estimates for incubation period, temperate/tropical strains. B) Kaplan-Meier estimates for time to first relapse, temperate/tropical strains (key in panel A). C) Kaplan-Meier estimates for incubation period, by region (key in panel F). D) Kaplan-Meier estimates for time to first relapse, by region (key in panel F). E) Flexible parametric survival model, incubation period projected for neurologic treatment–free populations, by region (key in panel F). F) Flexible parametric survival model, time to first relapse projected for neurologic treatment–free populations, by region.

The Kaplan-Meier plot of 447 case-patients who were included in the incubation period study shows a wide separation of Old World/New World groups by tropical/temperate region (Figure 1, panel A) and latitude/Hemisphere (Figure 1, panel C). The separations are statistically discernible by tropical/temperate region (log-rank test for equality χ^2^ = 127.9, 1 df, p<0.0001) and by latitude/hemisphere (log-rank test for equality χ^2^ = 204.6, 3 df, p<0.0001).

The unadjusted median incubation period for malaria caused by the combined tropical strains was 12 days (95% CI 12–12); that for the combined temperate strains was 15 days (95% CI 14–16). When stratified by Old World/New World, the tropical strains remain essentially unchanged, but a large separation occurred in the temperate strains: median survival was 14 days (95% CI 14–15) for New World temperate strains and 20 days (95% CI 19–21) for Old World temperate strains.

**Figure 2:**
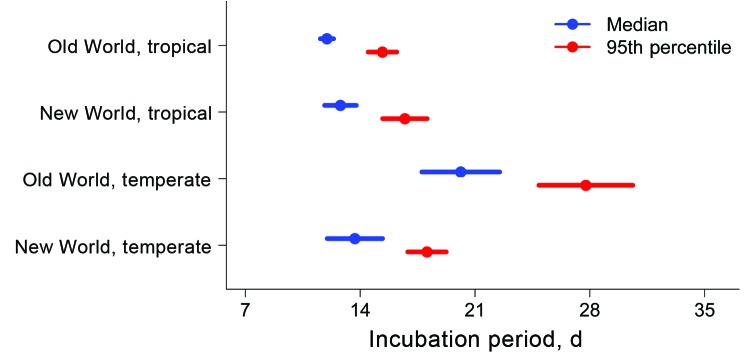
Length of incubation for *Plasmodium vivax* malaria infection, as determined by using flexible parametric survival models adjusted for neurologic treatment status, in a study quantifying the effect of geographic location on the epidemiology of the infection.

In the full multivariate model, after adjustment for neurologic treatment status, the median parametric survival estimate for the entire population was 13.6 days (95% CI 12.4–14.7) (Figure 1, panel E). The 95th percentile for the incubation period was 17.8 days (95% CI 16.6–18.9). Adjusted HRs differed between all regional categories, except for New World tropical/temperate categories, which did not achieve significance (p = 0.30). Predicted median and 95th percentile survival times are shown in Figure 2. Predicted median survival times were not different within the confidence intervals, except for Old World temperate strains (20.1 days [95% CI 17.8–22.5]); the 95th percentiles for temperate strains were significantly longer than those for tropical strains. HRs were 16.8 (95% CI 7.7–36.9) for the Old World, tropical region; 10.8 (95% CI 4.6–25.2) for the New World, tropical region; 7.3 (95% CI 3.8–14.0) for the New World, temperate region—all relative to the Old World, temperate region (reference). Persons infected with Old World tropical strains had a 16.8 (95% CI 7.7–36.9) times higher risk of clinical infection than did those with Old World temperate strains at each time point, leading to a shorter incubation period.

### Time to First Malaria Relapse

In all studies, the time to first relapse was measured from the reported primary infection. We used Kaplan-Meir survival curves to compare relapse times for the 316 persons included in these analyses and found large, statistically significant differences by tropical/temperate regions (log rank test for equality χ^2^ = 56.2, 1 df, p<0.0001) (Figure 1, panel B) and by latitude/hemisphere (log rank test for equality χ^2^ = 198.9, 3 df, p<0.0001) (Figure 1, panel D). Figure 1, panel D, shows that infections caused by the 2 Old World categories show shorter times to relapse compared with infections from New World parasites. In the full multivariate model (adjusted for neurologic treatment status), we found a distinct separation between the hemispheres (Figure 1, panel F); for the total population, we estimated the median time to relapse was 29.2 weeks (95% CI 25.0–33.4) and the 95th percentile was 61.4 weeks (95% CI 35.7–87.1).

**Figure 3:**
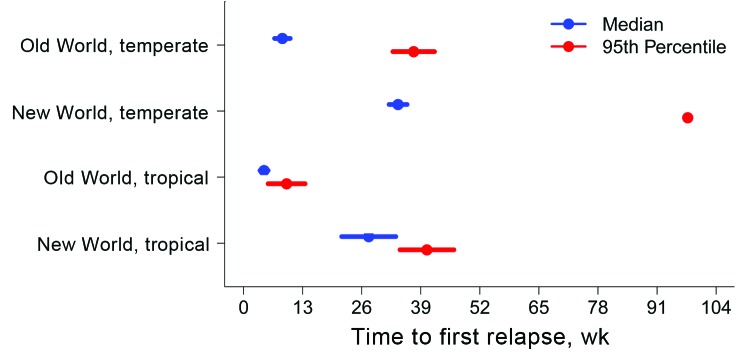
Predicted number of weeks from primary *Plasmodium vivax* malaria infection to first relapse, as determined by using flexible parametric survival models adjusted for neurologic treatment status, in a study quantifying the effect of geographic location on the epidemiology of the infection.

However, these aggregate values obscure substantial heterogeneity in time to relapse. Median relapse times for malaria caused by Old World parasites (tropical, 4.5 weeks [95% CI 3.6–5.4]; temperate, 8.5 weeks [95% CI 6.8–10.3]) were shorter than those for malaria caused by New World parasites (tropical, 27.5 weeks [95% CI 21.6–33.5]; temperate, 34.0 weeks [95% CI 32.0–36.0]). In addition, in both hemispheres, median relapse times for infections caused by tropical strains were shorter than those for infections caused by corresponding temperate strains, although this difference was not significant in the New World (Figure 3). The 95th percentile relapse times for the strain categories follow: Old World tropical, 9.5 weeks (95% CI 5.4–13.5); New World tropical, 40.3 weeks (95% CI 34.4–46.3); Old World temperate, 30.9 weeks (95% CI 19.9–41.9); and New World temperate, 97.7 weeks (95% CI 97.6–97.8). The HRs from the survival models (adjusted for neurologic treatment) follow: Old World tropical, 39.6 (95% CI 9.2–171.0; p<0.001); New World tropical, 0.93 (95% CI 0.36–2.41; p = 0.89); Old World temperate, 3.1 (95% CI 2.2–4.6; p<0.001)—all relative to New World temperate (reference).

### Distribution of Relapses

The total number of relapses was compared by latitude and hemisphere (Technical Appendix). An interval of 48 weeks was chosen to ensure equivalent follow-up periods between the regions (1-way analysis of variance *F*-test, follow-up time by region [p = 0.37]). To test the equality of distributions, we use the Kolmogorov-Smirnov test and found significant differences between tropical and temperate strains (p<0.001) and Old World and New World strains (p = 0.001). These differences remained significant when stratified by zones of latitude: temperate strains, Old World versus New World (p = 0.001); and tropical strains, Old World versus New World (p<0.001).

## Discussion

Malariologists have made a large number of observations concerning the geography-related epidemiology of *P. vivax* malaria (*22**,**23*). Malariotherapy treatments initially used a range of local strains from the United Kingdom, the Netherlands, and the United States, but these were quickly replaced with the Madagascar strain and others, which exhibited shorter incubation periods and more reliably produced infections (*24*). Estimates for the incubation period of *P. vivax* malaria vary: mean of 13.9 days (SD 3.7); 14 ± 3 days after the mosquito bite; 12–17 days (mean 15); and 8–17 days (*25*–*28*). A modern quantitative analysis of data for malariotherapy with a single strain (Madagascar) provided estimates of ≈10.3–16.9 days for the prepatent period (reported, in the study, to be generally 3 days longer than the incubation period) (*29*). After adjusting for neurologic treatment status, the estimated median from the parametric models for the population in our study is 13.6 days (95% CI 12.5–14.7), and the incubation period 95th percentile was 17.8 days (95% CI 16.6–18.9). The minimal differences between the Kaplan-Meir estimates and those from the multivariate models for incubation period strongly support earlier opinions that malariotherapy data are applicable to natural infections (*30*).

A range of strain-specific observations has also been reported for malaria relapses (*27*). Compared with persons with malaria caused by temperate strains, persons with malaria caused by tropical strains relapse more and have shorter relapses intervals (17–45 days), and a higher proportion have >2 relapses (*26*). It has been estimated that malaria caused by tropical strains relapses every 3–4 weeks, whereas malaria caused by temperate strains has longer, more variable periods between relapses (*31*).

Our finding suggests that these prior estimates for relapse intervals were based primarily on infections with Old World tropical strains. Our time-to-relapse estimates for other regions are considerably longer than estimates from earlier studies, with the exception of a study from El Salvador, which reported a median relapse interval of 28 weeks (*32*). The arithmetic median interval for malaria caused by all tropical strains (including only exact, non–interval censored times) was 20.0 weeks (95% CI 11.9–28.1). Results of the unadjusted Kaplan-Meier and parametric models, adjusted for neurologic treatment status in the full dataset, also indicate much longer median times to relapse: 20 weeks (95% CI 9–26) and 10.5 weeks (95% CI 3.7–17.4), respectively. Persons infected with Old World temperate strains have 39 (95% CI 9.2–171.0) times higher risk of relapse at each time point relative to those infected with New World temperate strains. Figure 1, panels D and F, suggests that the interaction between neurologic treatment and infection with different parasite strains has a substantial effect on the course of relapse; therefore, unadjusted relapse times from malariotherapy studies should be interpreted with caution.

In the data we studied, the number of relapses recorded within 48 weeks (for equivalent follow-up) is consistent with prior estimates: median of 2.6 relapses (95% CI 1.9–3.3; range 0–9) and of 0.68 relapses (95% CI 0.55–0.80; range 0–6) for case-patients with malaria caused by tropical and temperate strains, respectively. The data also showed that 68.5% (95% CI 64.9%–71.8%) of case-patients had relapses; this finding is broadly consistent with the previous finding that ≈60% of untreated cases relapse (*27*).

The general agreement of the number of relapses and incubation period among the population we studied with prior estimates suggests that the patients in our study do not represent a population substantially different from those with naturally acquired infections. However, the aggregate cohort values obscure large regional differences in epidemiology.

The effect of the geographic location of malaria parasites on the epidemiology of malaria has been long recognized: conspicuous differences in incubation period and disease latency have been broadly correlated with climatic zones (*27*). However, there have also been persistent difficulties in classifying these patterns; 2 different types of temperate strains (North American [St. Elizabeth] and European [Netherlands]) as well as tropical strains have been suggested (*14*). Inconsistencies have also been noted. For example, the tropical *P. vivax* strains in Central America show anomalous temperate zone epidemiology, leading to a suggestion that temperature alone might be an insufficient predictor of regional epidemiology (*33*). These conflicting observations are consistent with the results from this study (Figure 1, panel F), in which the most noticeable feature is that both categories of New World parasites caused malaria with substantially longer times to relapse, compared with malaria caused by Old World parasites.

Our findings suggest that the epidemiology of *P. vivax* infection has been occluded by inherent differences between parasite subpopulations and that hemisphere and latitude are strong drivers of the clinical manifestations and epidemiology of malaria. These findings strongly suggest that current paradigms for *P. vivax* clinical follow-up and surveillance may be based on erroneous assumptions. Malaria should not be discounted as a diagnosis even in the presence of long incubation periods, and the geographic origin of the parasite has critical effects on clinical features and should not be ignored in case histories.

Our results show that the mean incubation period for malaria caused by *P. vivax* strains from Eurasian temperate zones is statistically and clinically significantly longer than generally considered. This, plus the inherently longer extrinsic incubation period for malaria caused by Old World temperate strains, suggests that an active surveillance period of 31 days after potential exposure is the minimum necessary to capture the 95th percentile of new cases. However, this surveillance interval is balanced by a shorter median time to relapse for Old World temperate strains relative to all New World strains.

These 3 sets of independent measures (i.e., incubation period, time to first relapse, and distribution of the total patient relapses) across the entire course of illness suggest that *P. vivax* should not be considered a single parasite but is, in fact, several discrete and clinically distinct populations with unique and measurable characteristics. These data, plus previous entomologic and molecular evidence, support the delineation of subspecies within the range of the parasite: *P. vivax vivax* in the Eastern Hemisphere and *P. vivax collins* in the Western Hemisphere. This conclusion is supported by a recently published phylogenetic analysis of global *P. vivax* strains, which shows high diversity and clustering of isolates by hemisphere (*34*).

The origin of the infecting parasite affects the prophylaxis and treatment of malaria. Infections caused by strains from Korean Peninsula respond to standard doses of primaquine, but even higher doses did not fully suppress strains from New Guinea (Chesson), and infections caused by the Chesson strain required twice the dose of quinine relative to infections by a North American strain (McCoy) (*16*). Another related study also found large regional differences: infections with Thai strains were more likely to relapse and required higher primaquine dosing relative to infections from India or Brazil (*13*). The effect of malaria parasite population differences should be considered in the planning and analysis of interventional trials and in potential vaccine trials.

A recent analysis of malaria imported into the United States and Israel found that a large proportion of the case-patients exhibited long periods of latency: of 721 *P. vivax* case-patients with insufficient/nonexistent antimalarial drug prophylaxis, 46.5% (95% CI 42.8%–50.2%) had an incubation period >2 months, compared with 80.0% (95% CI 77.0%–82.8%) of case-patients with sufficient prophylaxis [authors’ calculations, from (*35*)]. However, no information was provided about the geographic source of these parasites, and the date of exposure is assumed to be the end of the travel period, making exact calculation of the incubation period impossible. The results from our study are broadly consistent with these values: 31.3% (95% CI 26.2%–36.6%) of case-patients in the time-to-relapse study had an incubation period >8 weeks. The comparability of these antimalarial drug–free populations suggests long-term stability in the global malaria parasite populations and also supports the relevance of these historical challenge studies for modern surveillance programs. The potential for prolonged incubation due to prophylaxis suggests that the estimates from our analysis should be considered as minimum values for travelers returning from vivax-endemic areas who had sufficient malaria prophylaxis.

Control and elimination programs for *P. vivax* should be reconsidered in light of these findings. Two major stumbling blocks identified during the First Global Malaria Eradication Campaign (1955–1972) were 1) the assumption that control methods could be universally applied and 2) burnout among program staff and funding agencies, resulting from continued surveillance at increasingly lower levels of infection (*36*). Addressing these issues in the current malaria elimination campaign will require detailed elucidation of the differences in pharmacodynamics among current parasite subpopulations and locale-specific malaria epidemiology, including estimation of incubation periods and the time to relapse to maximize surveillance efficiency.

Our results suggest that considerable complexity among *P. vivax* populations has been obscured by data aggregation; however, these divisions appear along defined geographic gradients. The long time interval of these studies (1920s–1980s) implies relatively stable parasite populations; however, the effect of greatly increased airplane travel, migration, and population-level antimalarial drug pressure should be explored.

The existence of subpopulations of *P. vivax* parasites along the division between the Eastern and Western Hemispheres enables conflicting historical and epidemiologic data to be formulated into a consistent and coherent picture, especially with the incorporation of phylogenetic approaches. In addition, parasite origin should be considered in drug prophylaxis and treatment, and the epidemiologic differences in disease caused by *P. vivax* subpopulations should be more fully elucidated.

Technical Appendix Detailed methods, models, comparison of the distribution of individual relapses, and primary literature references used to quantify the effect of geographic location on the epidemiology of *Plasmodium vivax* malaria.
