# Supplementary material for: Quantifying Effect of Geographic Location on Epidemiology of Plasmodium vivax Malaria
Source: Emerg Infect Dis. 2013 Jul;19(7):1058–65. doi: 10.3201/eid1907.121674 (PMC3713979; doi:10.3201/eid1907.121674)
Supplement: Technical Appendix — Detailed methods, models, comparison of the distribution of individual relapses, and primary literature references used to quantify the effect of geographic location on the epidemiology of Plasmodium vivax malaria. [file 12-1674-Techapp-s1.pdf]

Article DOI: http://dx.doi.org/10.3201/eid1907.121674

Quantifying Effect of Geographic Location on Epidemiology of *Plasmodium vivax* Malaria

Technical Appendix

I. Detailed Methods

All models were adjusted for neurologic treatment, as a binary variable; the predicted survival times have all been made for a neurologic treatment-free population (baseline predictions, using the –stpm2- predict –zeros- option). Intraclass correlation among parasite strain effects was modeled using robust standard errors via Stata’s vce(cluster clustvar) command (StataCorp, College Station, Texas, USA). The geographic regions were included in the time-to-relapse model as time-varying covariates due to extensive proportional hazard violations. The interaction between Old World, Temperate strains and neurologic treatment was significant and therefore included in the time-to-relapse model.

The proportional hazards assumption was tested using Schoenfeld residuals and by assessing the parallel nature of curves in log-log plots. Both the Akaike and Bayesian Information Criteria (AIC/BIC) were used to assess model parsimony and goodness-of-fit. Finally, for all models deviance residual plots were generated, and any significant outliers were identified and removed from the analysis. Goodness of fit was assessed by using a visual comparison of the fit models to Kaplan-Meier plots; the discriminatory power of the models was assessed with both Harrell’s c statistic and Somer’s D statistic, with bootstrapped errors. Statistical analysis was performed by using Stata 12.1; all tests were 2-tailed.

II. Models

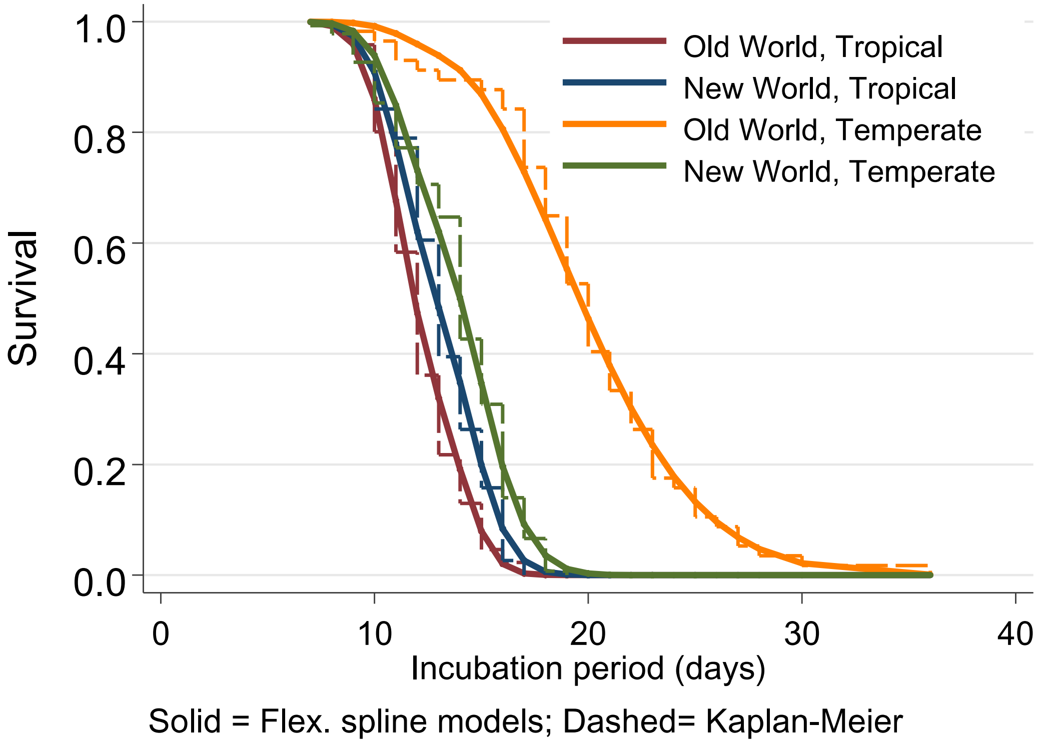

Technical Appendix Figure 1. Comparison of Kaplan-Meir estimates with Royston-Parmar flexible survival model, incubation period.

Technical Appendix Table 1. Royston-Parmar model, incubation period

Variable	Hazard ratio	95% CI	p value
Old World, Tropical	16.79	7.64–36.90	<0.001
New World, Tropical	10.77	4.60–25.24	<0.001
Old World, Temperate	Reference	–	–
New World, Temperate	7.30	3.82–13.96	<0.001
Neurotreatment	0.98	0.68–1.39	0.989

Harrell’s c statistic = 0.67 [bootstrapped 95% CI: 0.64-0.71].

Somer’s D statistic = 0.36 [bootstrapped 95% CI: 0.29–0.43].

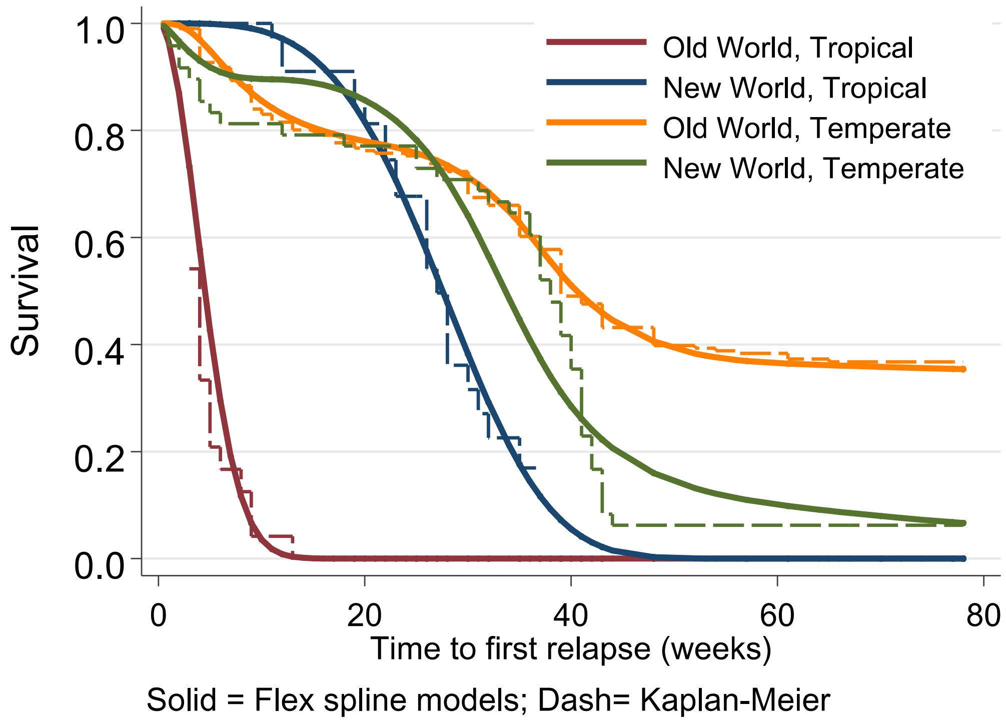

Technical Appendix Figure 2. Comparison of Kaplan-Meir estimates with Royston-Parmar flexible survival model, time-to-first relapse.

Technical Appendix Table 2. Royston-Parmar model, time-to-first relapse

Variable	HR	95% CI	P value
Old World, Tropical	39.60	9.17–170.91	<0.001
New World, Tropical	0.93	0.36–2.42	0.888
Old World, Temperate	3.15	2.18– 4.56	<0.001
New World, Temperate	Reference	–	–
Neurologic treatment	1.16	0.20–6.74	0.870
Old World, Temperate x neurologic treatment (interaction term)	0.15	0.024–0.92	0.040

Harrell’s c statistic = 0.85 [bootstrapped 95% CI: 0.76–0.93].

Somer’s D 0.69 [bootstrapped 95% CI: 0.54–0.84]

III. Distribution of Relapses

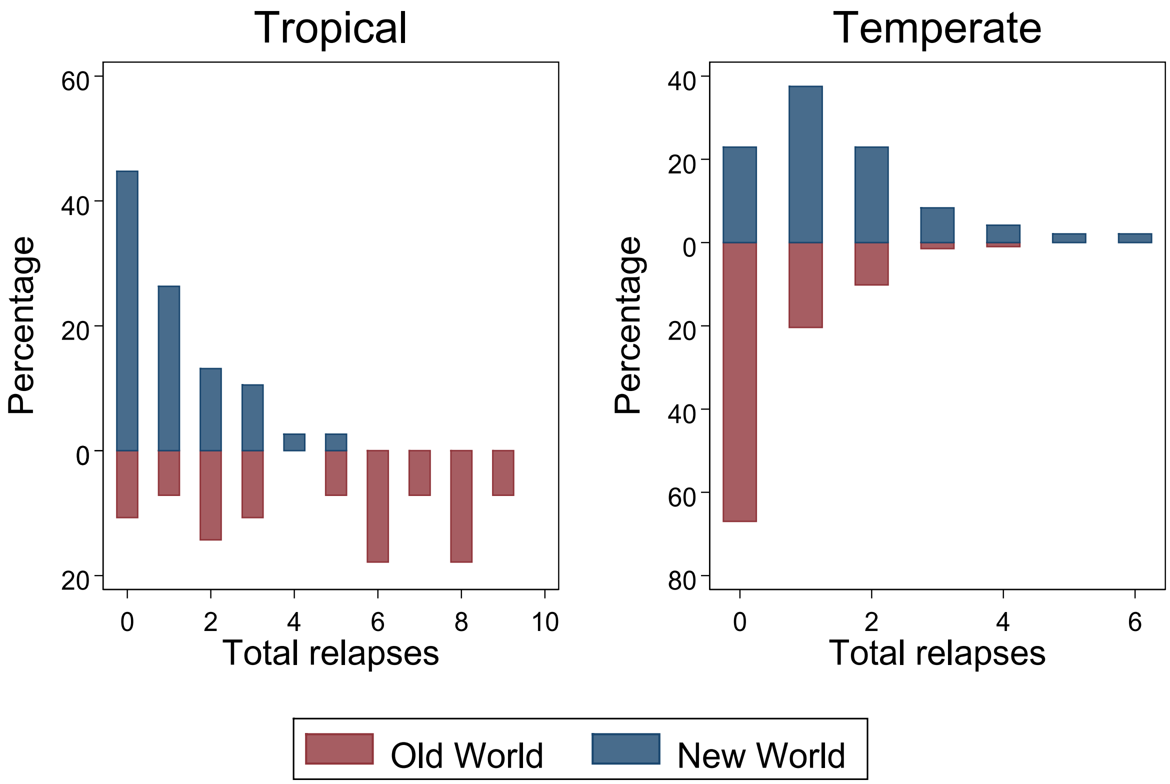

Technical Appendix Figure 3. Comparison of the distribution of total individual relapses within 48 weeks, by region.

IV. Primary Literature References

Technical Appendix Table 3. Primary literature references

Strain	Place, date of origin	Reference(s)
Chesson	Papua New Guinea, circa 1944	(*1–8*)
Hlebnikovo	Moscow Oblast, 1948	(*9*)
Holland	Netherlands, circa 1928	(*10,11*)
Korea	North Korea, 1953	(*9*)
Leninabad	Tajikistan, 1950	(*9*)
Madagascar	Madagascar, 1925	(*11–13*)
McCoy	Florida, USA, 1931	(*14*)
Moscow	Moscow, 1950	(*9*)
NICA	Nicaragua, circa 1970	(*15*)
Nahicevan	Azerbaijan, 1937	(*9*)
Naro-Fominsk	Moscow Oblast, 1946	(*9*)
Panama	Panama, circa 1970	(*15*)
Rjazan	Ryazan, Russia circa 1945	(*9*)
St. Elizabeth	South Carolina, USA, 1937	(*3,7,16–20*)
Salvador I	El Salvador, circa 1970	(*15*)
Salvador II	El Salvador, circa 1970	(*15*)
South Vietnam	Southern Vietnam, circa 1972	(*21*)
Vietnam (North)	Northern Vietnam, 1954	(*9*)
Volgograd	Volgograd, Russia, 1945	(*9*)
West Pakistan	Pakistan, 1968	(*22*)
P. vivax multinucleatum	Central China, 1965	(*23*)

References

1. Lints HA, Coatney GR, Cooper WC, Culwell WB, White WC, Eyles DE. Studies in human malaria XXII. Prolonged suppression of Chesson strain vivax malaria by the weekly administration of chlorguanide or chloroquine. J Natl Malar Soc. 1950;9:50–8.

2. Cooper WC, Coatney GR, Jeffery GM, Imboden CA. Studies in human malaria XXVIII. Observations on the use of chlorguanide against the Chesson strain of *Plasmodium vivax.* J Natl Malar Soc. 1950;9:366–76. PubMed

3. Cooper WC, Coatney GR, Culwell WB, Eyles DE, Young MD. Studies in human malaria XXVI. Simultaneous infection with the Chesson and the St. Elizabeth strains of *Plasmodium vivax.* J Natl Malar Soc. 1950;9:187–90. PubMed

4. Imboden CA, Cooper WC, Coatney GR, Jeffery GM. Studies in human malaria XXIX. Trials of aureomycin, chloramphenicol, penicillin, and dihydrostreptomycin against the Chesson strain of *Plasmodium vivax.* J Natl Malar Soc. 1950;9:377–80. PubMed

5. Coatney GR, Cooper WC, Young MD. Studies in human malaria XXX. A summary of 204 sporozoite-induced infections with the Chesson strain of *Plasmodium vivax.* J Natl Malar Soc. 1950;9:381–96. PubMed

6. Wiselogle FY, editor. A survey of antimalarial drugs 1941–1945. First edition. Ann Arbor (Michigan): J.W. Edwards; 1946.

7. Coatney GR, Ruhe DS, Cooper W, Josephson ES, Young MD. Studies in human malaria X. The protective and therapeutic action of chloroquine (SN 7618) against St. Elizabeth strain vivax malaria. Am J Hyg. 1949;49:49–59. PubMed

8. Coatney GR, Cooper WC, Eyles DE, Culwell WB, White WC, Lints HA. Studies in human malaria XXVII. Observations on the use of pentaquine in the prevention and treatment of Chesson strain vivax malaria. J Natl Malar Soc. 1950;9:222–33. PubMed

9. Tiburskaja NA, Sergiev PG, Vrublevskaja OS. Dates of onset of relapses and the duration of infection in induced tertian malaria with short and long incubation periods (WHO/Mal/67.604). 1967 [cited 2011 Dec 7]. http://whqlibdoc.who.int/malaria/WHO_Mal_67.604.pdf

10. Schüffner WAP, Korteweg PC, Swellengrebel NH. Experimental malaria with protracted incubation. Proc Koninklijke Nederlandse Akademie Wetenschappen. 1929;32:903–11.

11. Korteweg PC. Waarnemingen bij kunstmatig opgewekte malaria. Verschillende stammen van *Plasmodium vivax* [Observations in artificially induced malaria with two strains of *Plasmodium vivax*]. Ned Tijdschr Geneeskd. 1933;77(IV):4547–70.

12. Swellengrebel NH, De Buck A. Prophylactic use of plasmoquine in a dosage warranting reasonable safety for routine treatment. Proc Koninklijke Nederlandse Akademie Wetenschappen. 1931;34:1216–20.

13. Swellengrebel NH, De Buck A. Plasmoquine prophylaxis in benign tertian malaria. Proc Koninklijke Nederlandse Akademie Wetenschappen. 1932;35:912–4.

14. Putnam P, Boyd MF, Mead PA. Periodic or cyclically recurring phenomena of vivax malaria infections. Am J Hyg. 1947;46:212–47. PubMed

15. Contacos PG, Collins WE, Jeffery GM, Krotoski WA, Howard WA. Studies on the characterization of *Plasmodium vivax* strains from Central America. Am J Trop Med Hyg. 1972;21:707–12. PubMed

16. Coatney GR, Cooper CW, Ruhe DS, Josephson ES, Young MD, Burgess RW. Studies in human malaria VII. The protective and therapeutic action of quinine sulfate against St. Elizabeth strain vivax malaria. Am J Hyg. 1948;47:120–34. PubMed

17. Coatney GR, Cooper WC, Young MD, Burgess RW, Smarr RG. Studies in human malaria II. The suppressive action of sulfadiazine and sulfapyrazine against sporozoite-induced vivax malaria (St. Elizabeth strain). Am J Hyg. 1947;46:105–18. PubMed

18. Cooper WC, Ruhe DS, Coatney GR, Josephson ES, Young MD. Studies in human malaria VIII. The protective and therapeutic action of quinacrine against St. Elizabeth strain vivax malaria. Am J Hyg. 1949;49:25–40. PubMed

19. Coatney GR, Cooper WC, Young MD, Burgess RW. Studies in human malaria IV. The suppressive action of a phenanthrene amino alcohol, NIH-204 (SN-1796) against sporozoite-induced vivax malaria (St. Elizabeth strain). Am J Hyg. 1947;46:132–40. PubMed

20. Ruhe DS, Cooper WC, Coatney GR, Josephson ES. Studies in human malaria XII. The protective and therapeutic action of SN 5241 against St. Elizabeth strain vivax malaria. Am J Hyg. 1949;49:346–54. PubMed

21. Contacos PG, Collins WE, Chin W, Jeter MH, Briesch PE. Combined chloroquine-primaquine therapy against vivax malaria. Am J Trop Med Hyg. 1974;23:310–2. PubMed

22. Contacos PG, Coatney GR, Collins WE, Briesch PE, Jeter MH. Five day primaquine therapy—an evaluation of radical curative activity against vivax malaria infection. Am J Trop Med Hyg. 1973;22:693–5. PubMed

23. Jiang JB, Huang JC, Liang DS, Liu JX, Zhang SW, Cheng FC. Long incubation of *Plasmodium vivax multinucleatum* as demonstrated in three experimental human cases. Trans R Soc Trop Med Hyg. 1982;76:845–7. PubMed http://dx.doi.org/10.1016/0035-9203(82)90122-5
